# Helping Babies Breathe—Beyond Training

**DOI:** 10.9745/GHSP-D-18-00291

**Published:** 2018-10-03

**Authors:** Steve Hodgins

**Affiliations:** a Editor-in-Chief, Global Health: Science and Practice Journal, and Associate Professor, School of Public Health, University of Alberta, Edmonton, Alberta, Canada.

## Abstract

The revised Helping Babies Breathe training package now emphasizes the need for regular practice and quality improvement—an important improvement since more is needed than one-off training to have substantial impact on asphyxia-related newborn mortality.

See related articles by Kamath-Rayne.

## HELPING BABIES BREATHE, First Edition

The article by Kamath-Rayne et al.,^1^ in this issue of GHSP, reports on the review and revision of a widely deployed effort to reduce preventable asphyxia-related mortality and morbidity in newborns—Helping Babies Breathe (HBB). The American Academy of Pediatrics (AAP) developed this 1- to 2-day simulation-based training package on newborn resuscitation, finalizing it in 2010. Together with partners, including the World Health Organization, the United States Agency for International Development, Save the Children's Saving Newborn Lives program, the National Institute of Child Health and Human Development, and Laerdal Global Health, AAP subsequently introduced the program widely. The implementation effort, to date, has consisted primarily of large-scale campaigns to roll out the training (mainly on an in-service basis) and—in many cases—providing newborn resuscitation practice manikins in the health facilities where those trained are based. Impressively, close to half a million health workers across more than 80 countries have been reached with this training since HBB was first introduced.^2^

## PRACTICE, NOT ONLY TRAINING, IS NEEDED

As acknowledged by Kamath-Rayne et al., it is evident that even delivery of a well-designed training package is insufficient to reliably change clinical practice and improve outcomes. The article cites the 3 key elements of the “Utstein Formula for Survival” as *medical science, educational efficiency*, and *local implementation*. Under the first version of HBB, attention was directed primarily at the first 2 elements. Under the second edition of HBB,^3^ it has been better recognized that serious attention must also be directed to 2 aspects of the third element (local implementation): ongoing practice and quality improvement (Figure). This is an important step forward. As any musician or athlete is well aware, real skill mastery is not accomplished through just good training; it requires regular, serious practice. The same applies for skills essential for lifesaving clinical procedures like bag-and-mask resuscitation. And, with regular practice, comes not only skills but also the confidence to apply those skills when they're needed.

**FIGURE:**
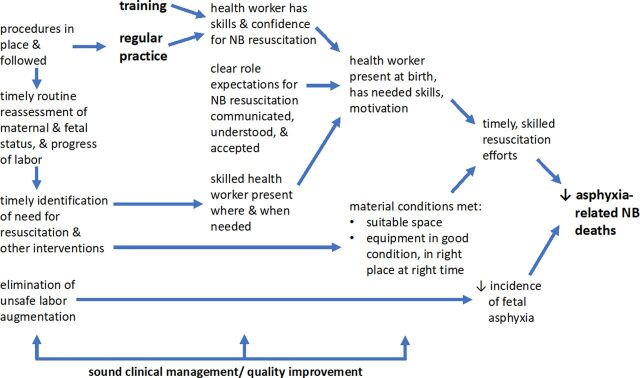
What Will It Take to Drive Down Asphyxia-Related Newborn Deaths?

## CLINICAL LEADERSHIP

It cannot be expected, however, that health workers will all, reliably and on their own initiative, regularly practice such skills. Clinical managers, notably those in charge of maternity services, must clearly communicate that, since asphyxia in the newborn is by far the most common life-threatening situation health workers will encounter at birth, it is the job of *every* health worker who could be present at birth to quickly and competently jump into action to resuscitate non-breathing newborns. And—to be reliably ready to do so—those who regularly attend births must regularly *practice* bag-and-mask resuscitation.

Since newborn asphyxia is by far the most common life-threatening situation health workers will encounter at birth, it is the job of *every* health worker who could be present at birth to quickly and competently jump into action to resuscitate non-breathing newborns.

Clinical managers also have a responsibility to ensure that:

- there are functional *staffing arrangements* in place, such that all births are reliably attended by health workers with the necessary skills (including newborn resuscitation), and
- the needed *material conditions* for timely and effective resuscitation are met, notably, availability of a suitable space in the immediate area where the birth takes place and immediate availability of all needed resuscitation equipment, in functional and clean condition.

## ANTICIPATING AND PREVENTING ASPHYXIA

As important as resuscitation is, our response to asphyxia-related morbidity and mortality in the newborn should not be confined to tertiary prevention, i.e., managing cases already potentially at death's door. There are important opportunities to intervene earlier in the causal process, with primary and secondary prevention strategies.

Cases of babies born asphyxiated and failing to spontaneously initiate breathing can usually be anticipated with regular, competent assessment of the laboring mother and her fetus. Abnormal fetal heart rate and meconium can signal fetal distress. With good labor management, problems can be picked up earlier and the health care team can mobilize to take timely action, ensuring that needed procedures are initiated on a timely basis.

In many clinical settings, labor is augmented using oxytocin, without adequate support to the mother, close monitoring of labor, and capacity to provide prompt emergency care. A predictable result is avoidable uterine hyperstimulation and fetal hypoxia, often with fatal consequences. Clinical managers responsible for childbirth care facilities have a responsibility to eliminate such unsafe practices.

## WHAT WILL IT TAKE TO MAKE A REAL DIFFERENCE?

HBB provides good hands-on training in newborn resuscitation. But rollout of a training package alone—even if it includes content emphasizing the importance of practice and quality improvement—cannot notably reduce asphyxia-related newborn morbidity and mortality. As recognized by Kamath-Rayne et al., and as reflected in the new HBB second edition materials, serious attention must also be directed at an array of implementation issues associated with timely, competent resuscitation of the non-breathing newborn. Furthermore, if it is our ambition to significantly reduce the burden of asphyxia-related newborn mortality, we must also give serious attention at earlier stages in the pathological process. Those working in the global newborn space who have resources and leadership responsibilities—take note.
